# Human umbilical cord mesenchymal stem cell exosome-derived miR-874-3p targeting RIPK1/PGAM5 attenuates kidney tubular epithelial cell damage

**DOI:** 10.1186/s11658-023-00425-0

**Published:** 2023-02-07

**Authors:** Yihang Yu, Meiling Chen, Qitong Guo, Lianju Shen, Xing Liu, Jianbo Pan, Yuanyuan Zhang, Tao Xu, Deying Zhang, Guanghui Wei

**Affiliations:** 1 Department of Urology, Children’s Hospital of Chongqing Medical University, Chongqing, 400014 China; 2 Chongqing Key Laboratory of Children Urogenital Department and Tissue Engineering, Ministry of Education Key Laboratory of Child Development and Disorders, China International Science and Technology Cooperation Base of Child Development and Critical Disorders, Chongqing, 400014 China; 3 National Clinical Research Center for Child Health and Disorders, Chongqing Key Laboratory of Pediatrics, Chongqing, 400014 China; 4 Center for Novel Target and Therapeutic Intervention, Institute of Life Sciences, Chongqing Medical University, Chongqing, 400016 China; 5 Wake Forest Institute for Regenerative Medicine, Wake Forest School of Medicine, Winston-Salem, NC 27101 USA; 6 Biomanufacturing Center, Department of Mechanical Engineering, Tsinghua University, Beijing, 100084 China

**Keywords:** Human umbilical cord mesenchymal stem cells, Exosome, Necroptosis, Mitochondrial fusion, miR-874-3p, Kidney tubular epithelial cell damage

## Abstract

**Background:**

Kidney insults due to various pathogenic factors, such as trauma, infection, and inflammation, can cause tubular epithelial cell injury and death, leading to acute kidney injury and the transformation of acute kidney injury to chronic kidney disease. There is no definitive treatment available. In previous studies, human umbilical cord mesenchymal stem cells have been shown to promote kidney injury. In this preclinical study, we investigate the role and mechanism of human umbilical cord mesenchymal stem cell exosomes (HucMSC-Exos) on the repair of renal tubular epithelial cells after injury.

**Methods:**

C57BL/6 mice underwent unilateral ureteral obstruction, and epithelial cell injury was induced in HK-2 cells by cisplatin. HucMSC-Exos were assessed in vivo and in vitro. The extent of renal cell injury, activation of necroptosis pathway, and mitochondrial quality-control-related factors were determined in different groups. We also analyzed the possible regulatory effector molecules in HucMSC-Exos by transcriptomics.

**Results:**

HucMSC-Exo inhibited necroptosis after renal tubular epithelial cell injury and promoted the dephosphorylation of the S637 site of the *Drp1* gene by reducing the expression of PGAM5. This subsequently inhibited mitochondrial fission and maintained mitochondrial functional homeostasis, mitigating renal injury and promoting repair. In addition, HucMSC-Exo displayed a regulatory role by targeting *RIPK1* through miR-874-3p.

**Conclusion:**

The collective findings of the present study demonstrate that HucMSC-Exos can regulate necroptosis through miR-874-3p to attenuate renal tubular epithelial cell injury and enhance repair, providing new therapeutic modalities and ideas for the treatment of AKI and the process of AKI to CKD transformation to mitigate renal damage.

**Supplementary Information:**

The online version contains supplementary material available at 10.1186/s11658-023-00425-0.

## Introduction

Owing to its unique microcirculatory characteristics and physiological functions, the kidney is one of the organs most vulnerable to ischemia, hypoxia, and toxic injury. Various external injuries, bacterial infections, and immune responses can frequently cause acute kidney injury (AKI). Patients with severe AKI have a poor prognosis. Most survivors gradually progress to chronic kidney disease (CKD) or end-stage renal disease (ESRD), requiring kidney transplantation or lifelong replacement therapy, which significantly reduces the quality of life and places a heavy burden on the patient’s family and society [1–4]. Renal fibrosis is a crucial link in the development of chronic transformation of various AKIs and CKDs and the most critical pathological basis for progression to ESRD [5]. In the pathological process of renal fibrosis, important links include tubular epithelial cell injury and death, inflammatory cell activation and aggregation, interstitial fibroblast proliferation, and extracellular matrix deposition [6, 7].

Necroptosis has recently been implicated as one of the most important pathways of renal tubular epithelial cell death in response to inflammatory stimuli [8]. Unlike highly coordinated and immunologically inert apoptosis, necroptosis is regarded as a passive and highly inflammatory form of cell death that results in the release of alarmins and other pro-inflammatory signals into the cellular surroundings [9]. Caspase8 is a crucial mediator of apoptosis and necroptosis [10]. Receptor-interacting protein kinase 1 (*RIPK1*) and receptor-interacting protein kinase 3 (*RIPK3*), which activates downstream mixed-lineage kinase domain-like pseudokinase (*MLKL*), is a critical regulatory pathway protein in necroptosis. *RIPK3*, as a sensor of environmental and cellular stress, is regulated by RIPK1 to initiate and deliver necrosis signals to *MLKL* and phosphoglycerate mutase 5 (*PGAM5*) [11, 12]. *PGAM5* is a serine/threonine protein phosphatase located in the outer mitochondrial membrane that binds to the necrosome vesicles formed by RIPK3–MLKL in the mitochondria and regulates mitochondrial division by promoting dephosphorylation of dynamin-related protein 1 (*Drp1*), causing an imbalance in mitochondrial quality-control homeostasis [13]. Mitochondrial reactive oxygen species (mtROS) and mitochondrial DNA (mtDNA) induce cellular necrosis [14]. In tumor necrosis factor (TNF)-induced cell necrosis, mtROS-driven autophosphorylation of RIPK1 is essential for the recruitment of RIPK3 to necrotic vesicles to cause necrosis [15]. Furthermore, the TNF-induced release of mtDNA into the cytoplasm activates DNA sensors, enhances RIPK3–MLKL-dependent necrosis, and promotes increased mitochondrial membrane permeability [16, 17].

We previously transplanted human umbilical cord mesenchymal stem cells (HucMSCs) into models of unilateral ureter obstruction (UUO) and aristolochic acid-induced renal fibrosis and renal tubular epithelial cell injury. The results showed that HucMSCs can effectively alleviate renal injury in model mice and significantly promote the repair of tubular epithelial cells [18–20]. However, allogeneic MSCs still have many limitations in clinical application due to potential immune rejection, tumor formation risk, and other aspects [21, 22]. As a recently discovered carrier of intercellular regulation, exosomes are the primary effector mode of the stem cell paracrine effect [23, 24]. Exosomes play an important role in the protection and repair of various tissue damages and can avoid the risks associated with stem cell therapy. With more apparent advantages, exosomes have become one of the most potent tools for the repair of tissue and organ damage. MicroRNAs (miRNAs) are currently considered the most diverse inclusions in exosomes. Experimentally, we discovered that miR-874-3p may be the principal miRNA regulating the repair of renal tubular epithelial cell injury in HucMSC-Exo. In this paper, we explored its relevance to necroptosis.

The present study was grounded on the hypothesis that HucMSC-derived exosomes (HucMSC-Exos) can regulate the occurrence of necroptosis through the delivery of miRNAs in the injured kidney, which is mainly regulated through miR-874-3p. Under the stimulation by pathological factors, the necroptosis pathway of renal tubular epithelial cells is activated as a key initiating factor that promotes kidney fibrosis. We assessed whether HucMSC-Exos inhibit RIPK1 through miR-874-3p to regulate the necroptosis pathway, maintain mitochondrial homeostasis, protect renal tubular epithelial cells, block the process of renal fibrosis, and promote renal injury repair.

## Materials and methods

### Experimental animals

Adult male specific-pathogen-free C57BL/6 mice (8–10 weeks old, 20–25 g) were purchased from Chongqing Medical University [license no. SYXK (Chongqing) 2007-0001]. The mice were raised in Chongqing Medical University Children’s Hospital Experimental Animal Center [license no. SYXK (Chongqing) 2007-0016]. The mice were housed in polycarbonate cages with a 12-h light/dark cycle at constant room temperature (24 °C) and controlled relative humidity (40–80%). All experimental procedures involving animals were conducted in accordance with the Basel Declaration and were approved by the ethics committee of Chongqing Medical University (no. CHCMU-IACUC20211222002).

### Isolation and identification of HucMSC-Exos

HucMSCs (cat.no. PCS-500-010) were provided by Chongqing Stem Cell Biotechnology R&D Base, Chongqing, China. The cells were cultured in Dulbecco’s modified Eagle medium nutrient mixture F-12 (DMEM/F12, Sigma-Aldrich, USA) containing 10% fetal bovine serum (Corning, USA) and 1% penicillin/streptomycin at 37 °C in a 5% CO_2_ atmosphere. The HucMSCs of`passage 1 to passage 6 are routinely cultured; when the cells reached around 80% confluence, the medium was replaced with an exosome-free serum medium, and the supernatant was collected after 48 h. The collected cell supernatant was centrifuged at low speed at 1000*g* for 10 min, then 2000*g* for 20 min, and finally at 10,000*g* for 30 min to remove cells and cell debris. The supernatant was filtered through a 0.22 μm filter and centrifuged at 100,000*g* for 70 min. The upper layer was discarded, and the lower layer was resuspended and concentrated by ultracentrifugation at 100,000*g* for 70 min. Finally, the exosomes were resuspended in PBS. A BCA kit was used to determine protein concentration in exosome preparations, and western blotting was used to assess exosome markers CD63, Alix, and Tsg101. One microliter of exosome suspension was taken, diluted 5000 times with PBS, resuspended, and mixed thoroughly. Then 20 μl of the diluted suspension was added dropwise to a copper mesh grid for an electron microscope, and it was let dry at room temperature overnight, fixed with 1% glutaraldehyde, and scanning electron microscopy was used to observe exosome morphology. The number and size of exosomes was directly tracked by the rate of Brownian motion of exosomes using ZetaView (Particle Metrix GmbH, Ammersee, Germany). The exosome suspension was diluted ten times with PBS and transferred to a cuvette; after 60 s of Brownian motion, the nanoparticle size and concentration distribution were determined. Besides, 2.5 μl of PKH26 was added to 200 μl exosome suspension and incubated at 37 °C for 5 min, then added the medium and centrifuged at 100,000*g* for 70 min, after which exosomes labeled with PKH26 could be obtained.

### Animals and groups

A mouse UUO model was established as previously described [19]. Twenty-four mice were randomly divided into four groups: sham, Exo, UUO, and UUO + Exo. Exos (100 µg) were injected through the tail vein 1 and 3 days after surgery. The kidneys were removed on day 7 after surgery; some were placed in paraformaldehyde, and some were placed at –80 °C for subsequent experiments.

### Cell culture and groups

HK-2 cells (cat. no. CRL-2190) purchased from the China Center for Type Culture Collection were cultured under the same conditions as used for HucMSCs. HK-2 cells grown to approximately 80–90% confluence were induced with cisplatin (5 μg/ml) in the absence or presence of HucMSC-Exo (100 μg/ml) with 24 h. Samples were collected after modeling for analyses.

### Histological observations

Kidney specimens were fixed in paraformaldehyde, dehydrated, paraffin-embedded, and sectioned to 4 μm thickness. After dewaxing and treatment with gradient alcohol, the slides were stained with hematoxylin–eosin (HE), periodic acid–Schiff (PAS), and Masson’s trichrome.

### Immunofluorescent staining

HK-2 cells were seeded in confocal culture dishes and incubated under the conditions described above. The cells were washed three times with PBS, fixed with 4% paraformaldehyde for 15 min each time, permeabilized with 0.2% Triton X-100 for 5 min, exposed to 0.5% bovine serum albumin (Solarbio, China) for 1 h, and then incubated with primary antibody overnight at 4 °C. The following day, the excess primary antibody was removed by washing three times with PBS, and the corresponding secondary antibody was added. Hoechst was used to stain nuclei of cells. Kidney tissue sections were dewaxed in water and placed in a citrate buffer solution for microwave heating to retrieve antigens. Endogenous peroxidase was blocked with 3% H_2_O_2_ for 15 min, after which the slides were washed three times with PBS. Following blocking with 5% bovine serum albumin for 1 h, the sections were incubated with primary antibody at 4 °C overnight. The subsequent fluorescence steps were the same as in the cell experiment. The images were obtained by a confocal microscope (Nikon, Japan) and were analyzed and processed by the Nis Viewer software.

### Immunohistochemistry

For immunohistochemistry, tissue sections were dewaxed in water and placed in citrate buffer solution for microwave heating to retrieve antigen. Endogenous peroxidase was blocked with 3% H_2_O_2_ for 15 min, after which the slides were washed three times with PBS. Following blocking with 5% bovine serum albumin for 1 h, the sections were incubated with primary antibody at 4 °C overnight. The corresponding secondary antibody (1:200 dilution; Zhongshan, China) was incubated for 1 h. Positive staining was achieved by incubation with 3,3′-diaminobenzidine (Zhongshan, China), and the sections were subsequently counterstained with hematoxylin. The images were examined by optical microscopy (Olympus, Japan).

### Real-time PCR

Total RNA was extracted from kidney tissues and HK-2 cells. The isolated RNA was reverse-transcribed using a miRcute Plus miRNA First-Strand cDNA Kit (Tiangen, China) according to the manufacturer’s instructions. Briefly, the samples were first added to lysate 1 (100 μl) and lysate 2 (600 μl), placed on the purification column, and centrifuged at 12,000 rpm for 30 s, then added the washing solution and centrifuged at 12,000 rpm for 30 s, three times; finally, DEPC water was added and centrifuged at 12,000 rpm for 30 s to obtain the total RNA. The quantity of miRNA was measured using SYBR Green I (Tiangen, China) and a CFX96 Real-Time PCR Detection System (Bio-Rad, USA). The relative expression levels of miR-874-3p were calculated by the comparative cycle threshold method using the expression of U6 small nuclear RNA as a reference for miRNA (sequence-specific primers are shown in Additional file 2: Table S1, S2). The 2^−ΔΔCt^ method was used for the analysis. The sequence-specific primers used for miR-874-3p and U6 are shown in Additional file 2.

### Western blot

HK-2 cells and kidney tissues were placed in RIPA lysis buffer (Beyotime Technology, China) with 1% phenylmethylsulfonyl fluoride to extract proteins. A BCA assay kit was used to measure the protein concentration. The proteins were subjected to SDS-PAGE and transferred onto polyvinylidene fluoride membranes (Millipore, USA). After blocking with blot blocking buffer (NCM, China) for 1 h, the membranes were incubated with various primary antibodies at 4 °C overnight (antibodies are shown in Additional file 2: Table S3), followed by three rinses with Tris-buffered saline/Tween, and incubated at a 1:5000 dilution of goat anti-mouse or goat anti-rabbit IgG secondary antibodies (Zenbio, China) for 1 h. Membranes were examined using enhanced chemiluminescence (ECL; Bio-Rad).

### Reactive oxygen species (ROS) and mitochondrial ROS (mtROS) detection

ROS production in HK-2 cells was detected using a ROS assay kit (Beyotime Technology). Briefly, live-HK-2 cells were incubated with 2′,7′-dichlorofluorescein diacetate at 37 °C for 20 min, washed with serum-free medium three times, and observed by fluorescence microscopy. Production of mtROS in HK-2 cells was detected using MitoSOX Red Mitochondrial Superoxide Indicator (Thermo Fisher Scientific, USA). Briefly, the MitoSOX reagent was added to Hank’s balanced salt solution (HBSS) to a concentration of 5 μM. The probe solution was loaded into viable HK-2 cells and incubated for 10 min at 37 °C, followed by three washes with HBSS, incubation for 10 min at 37 °C with Hoechst, washing three times with HBSS, and photography of fluorescence microscopy images.

### miRNA mimics/inhibitor transfection and dual-luciferase reporter assay

miR-874-3p mimics, miR-874-3p inhibitor, and corresponding negative controls were obtained from RiboBio Company (China). HK-2 cells were seeded in six-well plates and transfected with Lipofectamine 3000 (Invitrogen, USA), according to the manufacturer’s instructions. The construction of recombinant plasmids in the 3′-untranslated region (UTR) of the RIPK1 gene, wild type (h-RIPK1-WT), and mutant (h-RIPK1-MUT) was performed by the RiboBio Company. Co-transfection of recombinant plasmids and miR-874-3p mimics and miR-874-3p NC mimics was performed in HK-2 cells. According to the manufacturer’s instructions, firefly and Renilla luciferase activities were measured 48 h after co-transfection using a Dual-Luciferase Reporter Assay Kit (Promega, USA).

### Co-immunoprecipitation (Co-IP) analysis

Co-IP was performed using a Co-IP assay kit (Absin, China). Protein lysates from HK-2 cells were incubated with a PGAM5 antibody overnight at 4 °C. Immune complexes were bound to protein A/G agarose beads. The complexes were centrifuged and resuspended in SDS, and western blotting was used to detect protein expression. Normal IgG was used as a negative control.

### Statistical analyses

Experimental data are expressed as mean ± standard deviation (SD). Statistical analyses were performed using one-way analysis of variance (ANOVA) for comparison, followed by Dunnett’s post-hoc test. All data were analyzed using GraphPad Prism 8.0 (GraphPad Software, USA), and *p*-values < 0.05 were considered statistically significant.

## Results

### Identification and labeling of HucMSC-Exos

**Fig. 1:**
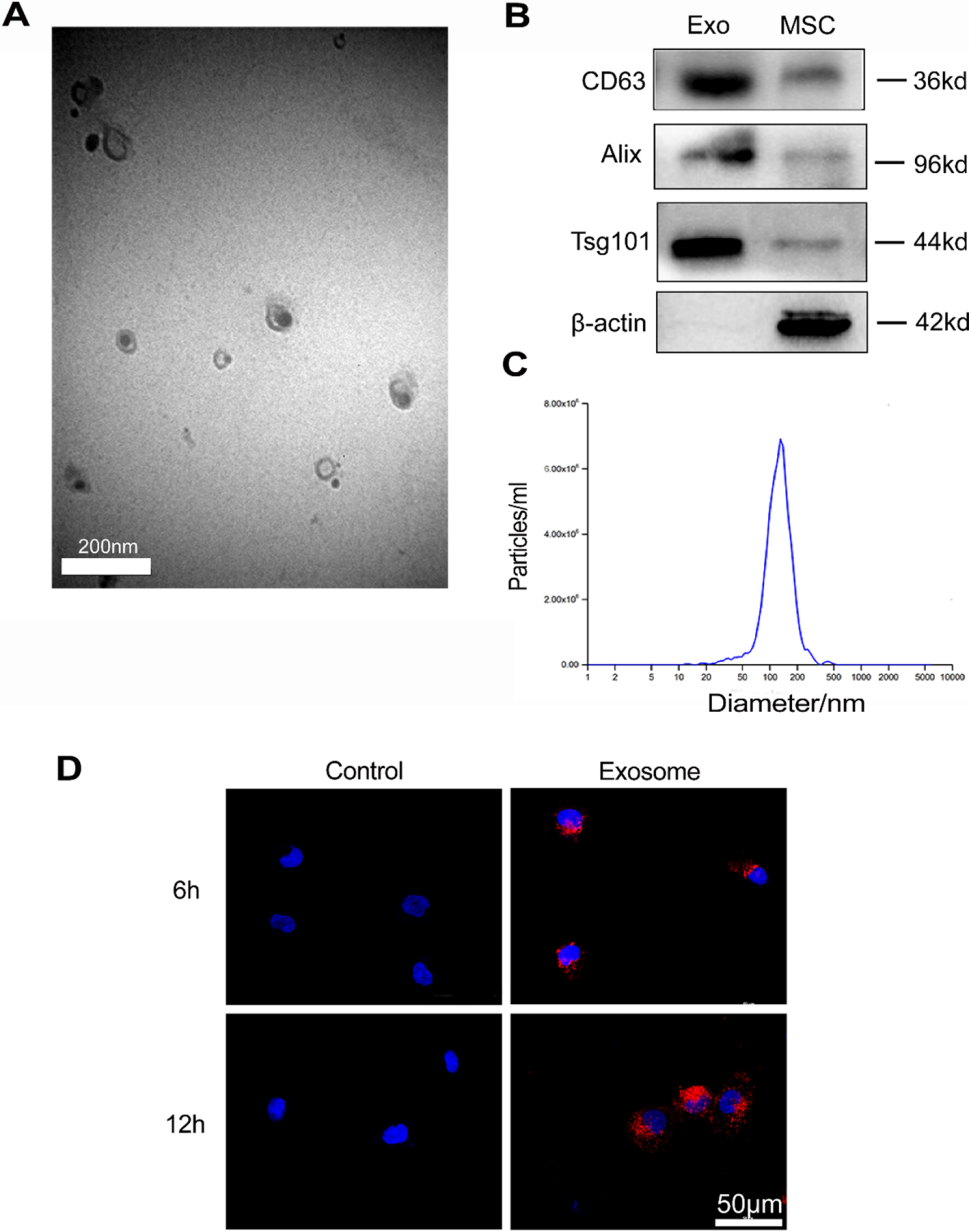
Identification and labeling of HucMSC-Exo. **A** Morphology of HucMSC-Exo detected by electron microscopy. **B** Western blot detection of exosome-related markers in HucMSC-Exo and MSC. **C** HucMSC-Exo nanoparticle size analysis detected by Particle Metrix’ ZetaView; particles were mainly enriched at 128.7 nm. **D** HK-2 cell uptake of HucMSC-Exo labeled by PKH-26

To determine whether exosome extraction was successful, we observed preparations by electron microscopy. Round or oval exosome vesicles with diameters ranging from 30 to 150 nm and a bilayer membrane structure were evident. Nanoparticle size analysis revealed that the particles were mainly enriched at 128.7 nm (Fig. 1A and C). Western blotting detected the CD63, Alix, and Tsg101 exosome markers (Fig. 1B). These findings demonstrated the successful extraction of exosomes from the HucMSC supernatant. We examined the uptake of PKH-26-labeled exosomes by HK-2 cells in vitro. Fluorescence microscopy revealed the uptake of exosomes by HK-2 cells after 6 and 12 h (Fig. 1D). The findings supported the use of a 6-h pretreatment time in subsequent in vitro experiments.

### HucMSC-Exos improve renal pathological structure and fibrosis in UUO

**Fig. 2:**
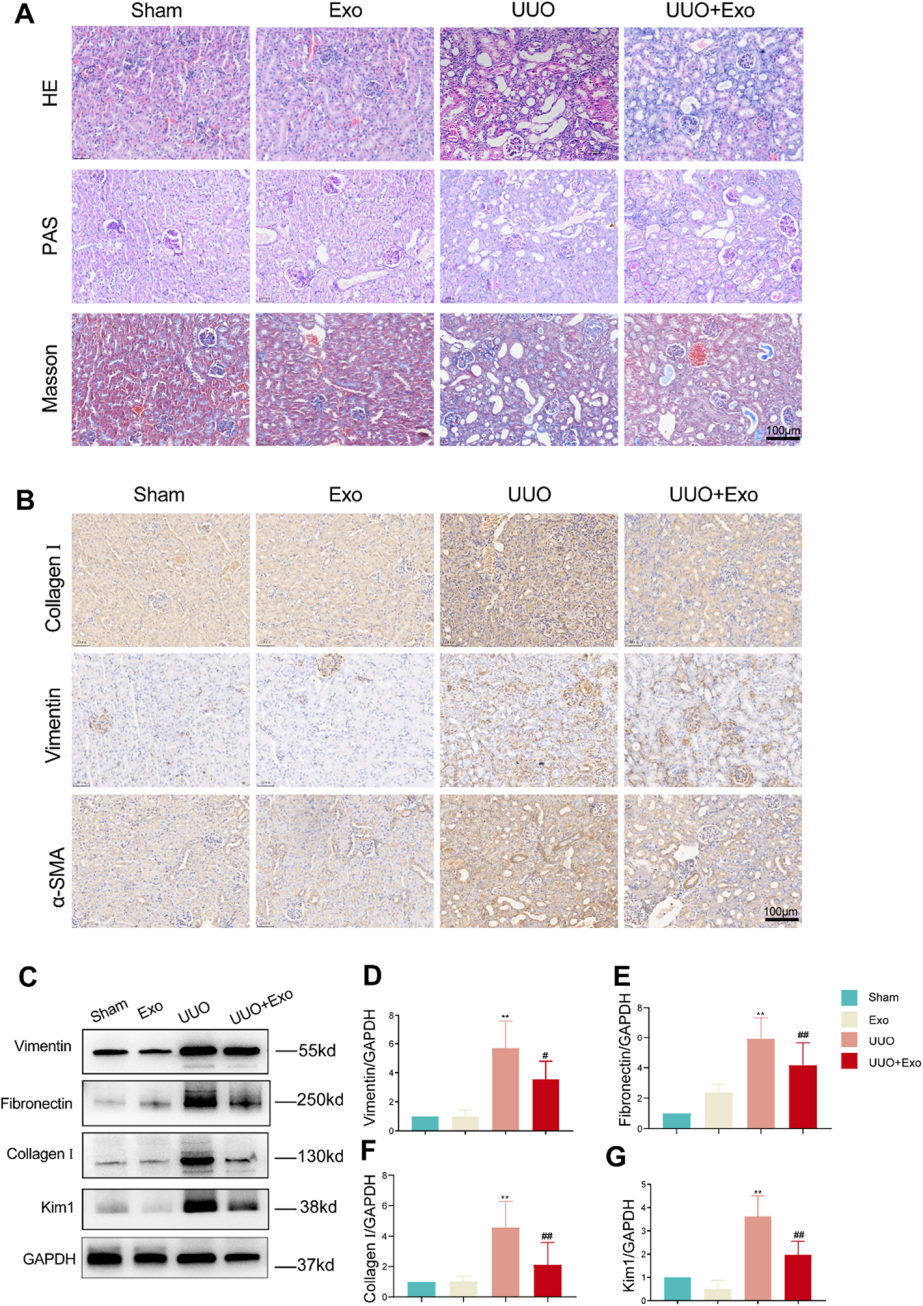
Examination of kidney morphology and fibrosis markers in four groups in vivo. **A** HE, PAS, and Masson staining in each group. **B** Immunohistochemistry of fibrosis-related markers (Collagen I, Vimentin, and α-SMA) in each group. **C** Western blot of fibrosis-related markers (Collagen I, Vimentin, and α-SMA) and Kim1 in each group. **D**–**G** Relative expression of fibrosis-related markers and Kim1 detected by western blot. *p < 0.05, **p < 0.01, compared with sham group; ^#^*p* < 0.05, ^##^*p* < 0.01, compared with UUO group

To observe the morphological changes in the kidneys of mice treated with UUO and HucMSC-Exos, kidney sections were examined with HE and PAS staining (Fig. 2A). The morphology of the kidneys in the Sham and Exo groups was intact with no significant abnormalities. However, in the UUO group, renal tubular dilatation or atrophy with loss of brush border and massive inflammatory cell infiltration with disorganization of the interstitial region were observed. Furthermore, the number of sites and severity of renal structural damage was significantly less than that in the UUO group after intervention with HucMSC-Exos. Masson’s trichrome staining revealed the deposition of many collagen fibers in the interstitial region of the UUO group (Fig. 2A). In contrast, the area of fiber deposition was significantly reduced in the UUO + Exo group compared with that in the UUO group.

In addition, we examined the expression of fibrosis-related markers, vimentin, fibronectin, and collagen I, and the tubular epithelial injury marker, kidney injury molecule 1 (Kim1), in various groups in vivo. Western blotting revealed that the expression of all of these indicators was significantly increased after UUO, and exosome intervention (Fig. 2C) effectively suppressed the expression of these factors. The immunohistochemistry results were consistent with the western blotting results (Fig. 2B). These findings demonstrate that HucMSC-Exo intervention can effectively alleviate the progression of renal fibrosis and tubular epithelial cell injury in vivo and promote kidney repair after injury.

### HucMSC-Exos inhibit necroptosis pathway in vivo

**Fig. 3:**
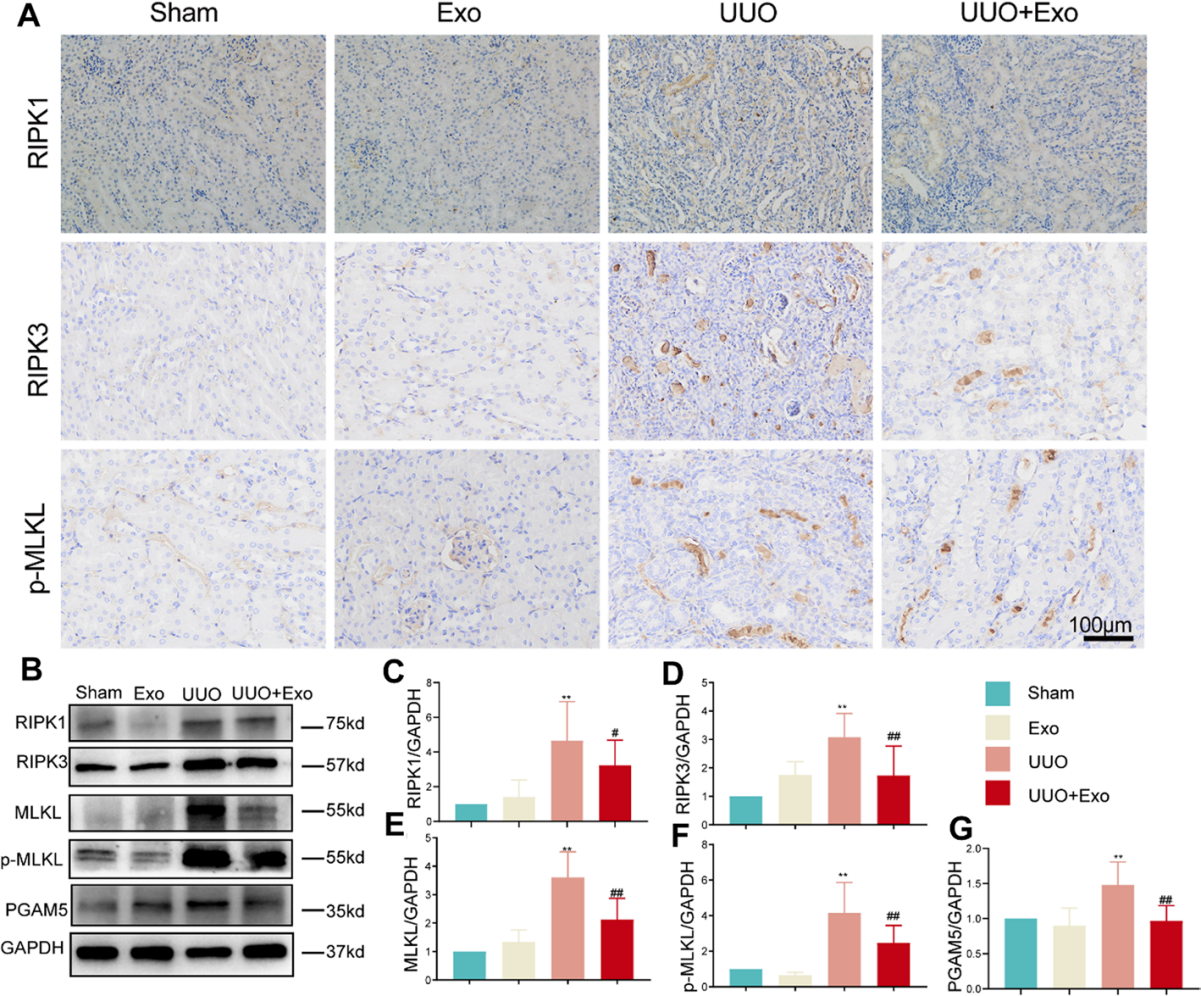
Examination of necroptosis-related markers in four groups in vivo. **A** Immunohistochemistry detected of necroptosis-related markers (RIPK1, RIPK3, and p-MLKL) in each group. **B** Western blot of necroptosis-related markers (RIPK1, RIPK3, MLKL, p-MLKL, and PGAM5) in each group. **C**–**G** Relative expression of necroptosis-related markers detected by western blot. *p < 0.05, **p < 0.01, compared with sham group; ^#^*p* < 0.05, ^##^*p* < 0.01, compared with UUO group

The expression of RIPK1, RIPK3, and p-MLKL proteins related to the necroptosis pathway were significantly increased in exosome-injected UUO-modeled mice (Fig. 3A), suggesting that the necroptosis pathway was activated, and necroptosis pathway-related proteins were inhibited after exosome intervention. The results obtained by western blotting and immunohistochemistry were consistent (Fig. 3B–G). Previous research has shown a critical regulatory role of PGAM5 in cell death mediated by necroptosis [25]. We further detected PGAM5 expression in vivo. The expression trend of PGAM5 was the same as that of necroptosis-related proteins. The experimental results demonstrated that the necroptosis pathway was activated when kidney cells were damaged, further promoting the expression of PGAM5.

### HucMSC-Exos promote mitochondrial fusion in vivo

**Fig. 4:**
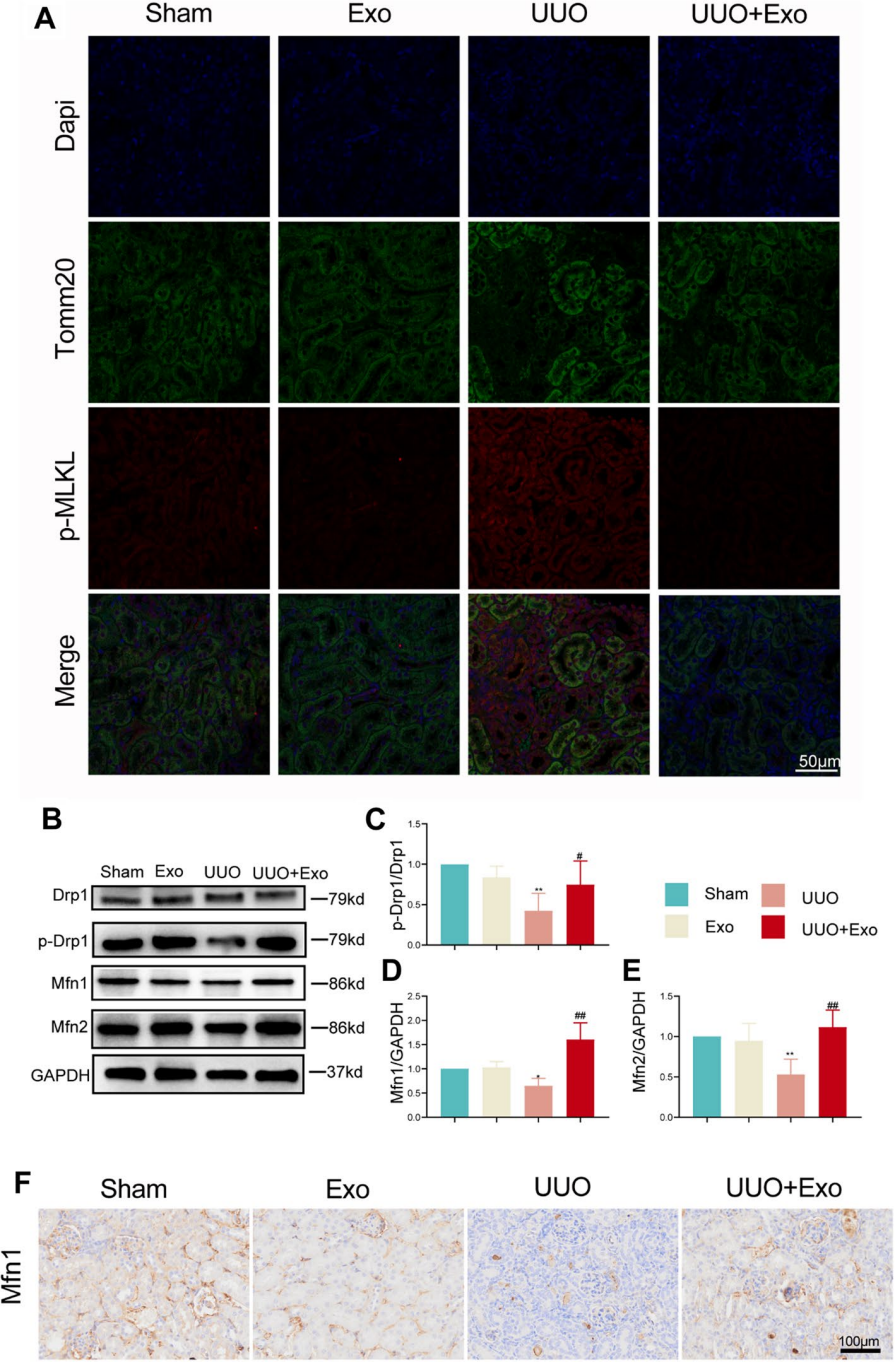
Examination of mitochondrially related markers in four groups in vivo. **A** Immunofluorescence of Tomm20 and p-MLKL in the kidney sections in four groups. **B** Western blot of mitochondrial fusion-related markers (Drp1, p-Drp1, Mfn1, and Mfn2) in each group. **C**–**E** Relative expression of mitochondrial fusion-related markers detected by western blot. *p < 0.05, **p < 0.01, compared with sham group; ^#^*p* < 0.05, ^##^*p* < 0.01, compared with UUO group. **F** Immunohistochemistry of Mfn2 in each group

Double immunofluorescence staining revealed that distinct mitochondrial membrane structures (translocase of the outer mitochondrial membrane complex subunit 20, Tomm20) were visible in renal tubules in the sham group (Fig. 4A). In contrast, in the UUO group, mitochondrial membrane structures were not visible in renal tubules with deposition of necroptosis markers (phosphorylated MLKL and p-MLKL), suggesting disruption of the mitochondrial structure. We hypothesized that there is interactive regulation of necroptosis and mitochondrial quality control. Therefore, we next examined the expression of the mitochondrial kinetic protein Drp1 in vivo. Increased Drp1 dephosphorylation was evident in the UUO group, and decreased phosphorylated Drp1 expression further caused mitochondrial quality control disorder. Therefore, we examined the expression of mitochondrial fusion proteins mitofusin (Mfn)1 and Mfn2 (Fig. 4B and F); their expression was reduced in the UUO group. This trend was reversed by the addition of exosomes. These results demonstrate that the intervention of HucMSC-Exos can effectively inhibit the promotion of mitochondrial fission in UUO-modeled injury.

### HucMSC-Exos inhibit the necroptosis pathway and ROS production in vitro

**Fig. 5:**
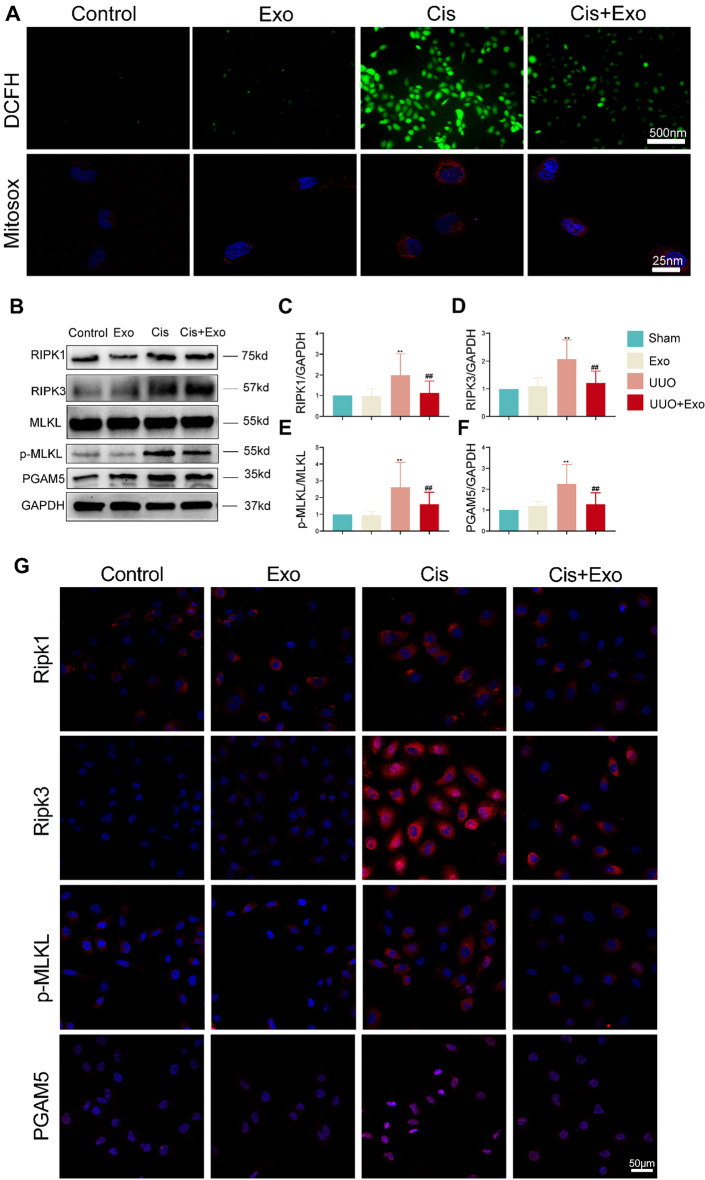
Examination of ROS level and necroptosis-related markers in four groups in vitro. **A** Immunofluorescence detected with the ROS indicator DCFH (green) and the mitochondrial ROS (mtROS) indicator MitoSox (red) in each group. **B** Western blot of necroptosis-related markers (RIPK1, RIPK3, MLKL, p-MLKL, and PGAM5) in each group. **C**–**F** Relative expression of necroptosis-related markers detected by western blot. *p < 0.05, **p < 0.01, compared with control group; ^#^*p* < 0.05, ^##^*p* < 0.01, compared with cisplatin (Cis) group. **G** Immunohistochemistry of necroptosis-related markers in each group

In vitro, we added exosomes to the culture medium 6 h before pretreatment, followed by the addition of cisplatin to induce renal tubular epithelial cell injury. The results showed that cisplatin-induced necroptosis pathway protein expression was significantly increased in HK-2 cells, whereas it significantly decreased in HK-2 cells pretreated with exosomes compared with the cisplatin group (Fig. 5B and G, Additional file 1: Fig. S2A–D).

In addition, we examined the expression of ROS and mtROS in different groups of HK-2 cells (Fig. 5A). The expression of ROS and mtROS was significantly increased in the cisplatin-induced HK-2 injury model. Simultaneously, ROS production appeared to be decreased considerably after exosome intervention. ROS production was directly correlated with necroptosis, and mtROS, a byproduct of mitochondrial respiration, was significantly increased by cisplatin injury in HK-2 cells, after which mtROS expression was significantly reduced.

### HucMSC-Exos promote mitochondrial fusion by inhibiting PGAM5 dephosphorylation of Drp1 in vitro

**Fig. 6:**
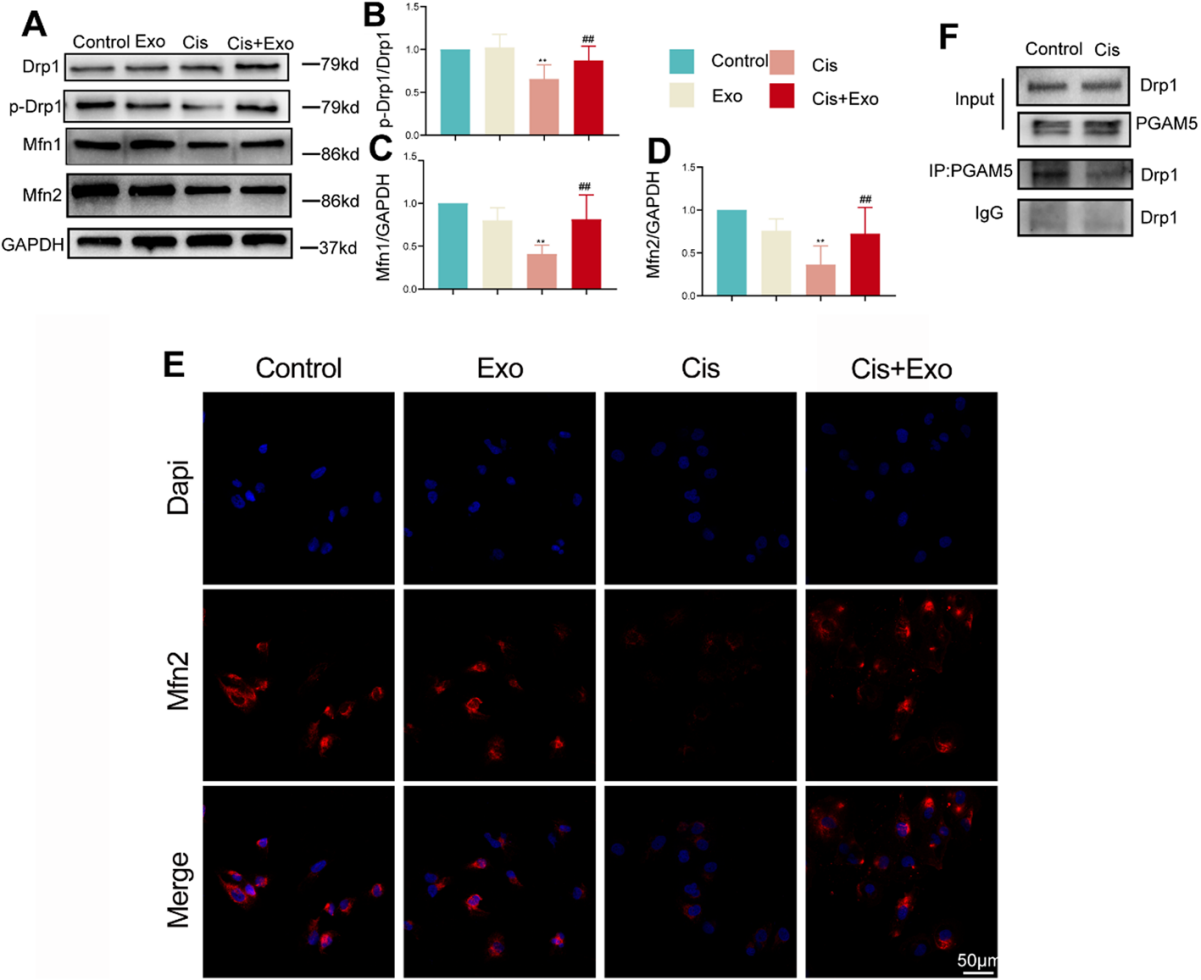
Examination of mitochondrially related markers in four groups in vitro. **A** Western blot of mitochondrial fusion-related markers (Drp1, p-Drp1, Mfn1, and Mfn2) in each group. **B**–**D** Relative expression of mitochondrial fusion-related markers detected by western blot. *p < 0.05, **p < 0.01, compared with control group; ^#^*p* < 0.05, ^##^*p* < 0.01, compared with cisplatin (Cis) group. **E** Immunohistochemistry of Mfn2 in each group. **F** Co-immunoprecipitation assays detecting Drp1 and PGAM5 interaction in HK-2 cells

Next, we examined the expression of mitochondrial dynamin protein Drp1 in vitro. Exosomes effectively inhibited cisplatin-induced dephosphorylation of Drp1. Significant increases in the expression of mitochondrial fusion proteins Mfn1 and Mfn2 were observed after exosome intervention compared with that in the cisplatin group (Fig. 6A and E). In addition, we detected endogenous interactions between PGAM5 and Drp1 in HK-2 cells using Co-IP assays (Fig. 6F). The collective data indicate that PGAM5 acts as an upstream mediator of Drp1S637 dephosphorylation to promote cisplatin mitochondrial fission. The findings also demonstrated in both in vivo and in vitro examination that HucMSC-Exos can inhibit the activation of necroptosis and abnormalities in mitochondrial fission.

### HucMSC-Exo-derived miR-874-3p targeting of RIPK1 inhibits necroptosis

**Fig. 7:**
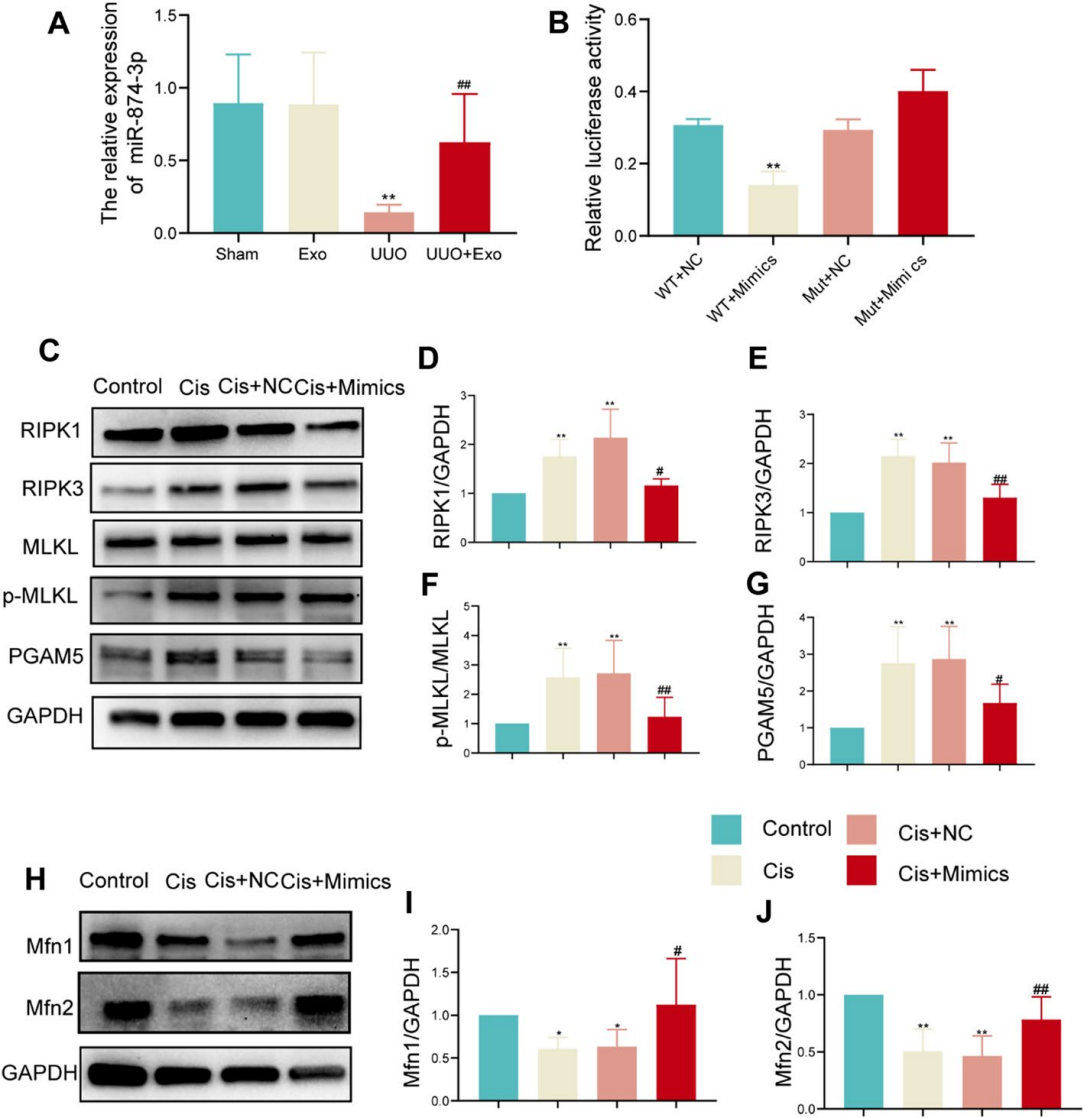
Detection of miR-874-3p expression and its targeting to necroptosis. **A** Quantitative PCR detects the expression of miR-874-3p in each group in vivo. **p < 0.01, compared with sham group; ^##^*p* < 0.01, compared with UUO group. **B** Detection of miR-874-3p targeting RIPK1 by dual-luciferase reporter gene assay. **p < 0.01, compared with WT + NC group. **C** Western blot of necroptosis-related markers (RIPK1, RIPK3, MLKL, p-MLKL, and PGAM5) in each group. **D**–**G** Relative expression of necroptosis-related markers detected by western blot. *p < 0.05, **p < 0.01, compared with control group; ^#^*p* < 0.05, ^##^*p* < 0.01, compared with cisplatin (Cis) group. **H** Western blot of Mfn1 and Mfn2 in each group. **I**, **J** Relative expression of Mfn1 and Mfn2 detected by western blot. *p < 0.05, **p < 0.01, compared with control group; ^#^*p* < 0.05, ^##^*p* < 0.01, compared with Cis group

The preceding experiments demonstrated that exosomes could further influence mitochondrial quality control by regulating the necroptosis pathway, thereby promoting mitochondrial homeostasis and damage repair in renal tubular epithelial cells. Next, we screened HucMSC-Exos as a possible key effector of miR-874-3p by comprehensive analysis of miRNA sequencing of renal tissue in the sham-operated and UUO groups (Additional file 1: Fig. S1A). The analysis indicated the targeting of RIPK1, a critical molecule of necroptosis (Additional file 1: Fig. S1B and C). Furthermore, miR-874-3p could target and regulate the expression of RIPK1. We examined the expression of miR-874-3p in various groups in vivo and found that its expression was the lowest in the UUO group, while there was an increase in its expression after exosome intervention (Fig. 7A).

We successfully transfected miR-874-3p into HK-2 cells in vitro and demonstrated that miR-874-3p could target RIPK1 using a dual-luciferase reporter gene assay (Fig. 7B and Additional file 1: Fig. S1D). In addition, we transfected mimics/NC with miR-874-3p in HK-2 cells and added cisplatin to investigate whether miR-874-3p regulates the development of necroptosis (Fig. 7C). Cisplatin-induced necroptosis was significantly inhibited by the addition of miR-874-3p mimics, while there was no significant difference between the NC and cisplatin addition groups. Further detection of mitochondrial fusion proteins (Fig. 7H) and the expression of Mfn1 and Mfn2 revealed inhibition in the mimics NC and cisplatin addition groups and significantly increased expression in the Cis + mimic group.

## Discussion

In this study, we identified the role of HucMSC-Exos in promoting the repair of renal tubular epithelial cell injury. Our results suggest five points: First, tubular epithelial cells undergo oxidative stress and necroptosis after kidney injury and play a critical regulatory role. Second, PGAM5 can further aggravate tubular epithelial cell injury after necroptosis by dephosphorylating the S637 site of Drp1, thereby unbalancing mitochondrial quality-control homeostasis, promoting mitochondrial division, and reducing mitochondrial fusion. Third, intervention using HucMSC-Exos can effectively reduce the extent of renal tubular epithelial cell injury and promote post-injury repair. Fourth, HucMSC-Exos can promote renal tubular epithelial cell repair by inhibiting necroptosis and restoring mitochondrial homeostasis. Finally, HucMSC-Exo-derived miRNA may target *RIPK1*, with miR-874-3p having a role in regulating programmed necrosis and mitochondrial division. Mechanistically, intervention using HucMSC-Exos can attenuate oxidative stress after renal tubular epithelial cell injury, reduce the activation of the necroptosis pathway, promote homeostasis of mitochondrial quality control, and successfully promote repair of tubular epithelial cell injury.

Apoptosis may be important in the progression of AKI. Inhibiting apoptosis can effectively block the progression of AKI and promote renal repair [26]. Nevertheless, recent studies have found that inhibitors of caspases failed to protect renal function in mice with ischemia–reperfusion injury. Additionally, deleting apoptosis-related proteins in the renal tubules, such as caspase8 and Fas-associated protein with death domain, did not protect against AKI [27, 28]. On the basis of the above studies, many researchers believe that apoptosis is not crucial for AKI [29]. Other forms of cell death occur during kidney injury. Necroptosis is a regulated, pro-inflammatory, and caspase-independent form of necrotic cell death [30]. TNF receptor engagement leads to the activation of RIPK1 and RIPK3, leading to the formation of the necrosome. RIPK3 phosphorylates MLKL, leading to necroptosis activation. Active oligomerized MLKL mediates plasma membrane rupture, killing the cell [13, 31, 32]. Aberrant levels of necroptosis have been implicated in various inflammatory diseases and ischemic injuries, making this cell death mechanism an important therapeutic target [33]. Necroptosis has an essential regulatory role in AKI and renal transplantation [34–37]. Activation of mtROS in some cells enables autophosphorylation of RIPK1, leading to the activation of necroptosis pathways and the formation of the necrosome [15, 38]. RIPK3 activates the pyruvate dehydrogenase complex in a feed-forward manner, contributing to enhanced aerobic respiration and mtROS generation [39]. Increased levels of ROS can promote the opening of the mitochondrial permeability transition pore and affect mitochondrial dysfunction, leading to an increased likelihood of cell necrosis [40, 41]. Mitochondrial homeostatic imbalance and necroptosis form a feedback loop that further aggravates cellular damage. Therefore, we focused on necroptosis in kidney cell injury.

HucMSCs could be an ideal therapeutic approach because of their beneficial properties, including easy extraction and expansion, low cost, noninvasive collection procedures, significant cell content, low risk of infection, high proliferation factors, and low immunogenicity [42, 43]. Therefore, we selected HucMSCs as the seed cells of exosome origin. Compared with their parent cells, MSC-Exos have a better safety profile and can be stored without losing function [44]. They have been considered in recent years as ideal vesicles for future biologic therapies and have demonstrated their role and potential in the repair of damage in a variety of organ systems [23, 45–47]. Exosomes are EVs with a size range of 40–160 nm (average ~ 100 nm) in diameter and exert their regulatory effects mainly through their inclusions, such as proteins, miRNAs, and long noncoding RNAs [48–50]. Of these, miRNAs are currently considered the most diverse. miR-874-3p reportedly inhibits tumor growth or metastasis by regulating signal transducer and activator of transcription 3, cyclin-dependent kinase 9, and E2F transcription factor 3; reduces brain ischemia by regulating BCL-2-modifying factor in reperfusion injury; and reduces apoptosis and inflammation in alveolar epithelial cells by targeting early growth response 3/nuclear factor-kappa B [51–53]. According to previous experiments, miR-874-3p inhibits tumor metastasis and apoptosis to promote tissue environment repair and homeostasis. In the present study, the correlation between miR-874-3p and necroptosis was discovered for the first time. miR-874-3p regulated necroptosis and mitochondrial fission in renal tubular epithelial cells by targeting RIPK1 to promote injury repair in the kidney.

Exosomes derived from MSCs are very promising for the treatment of various diseases. Yet, the clinical application of MSC-Exos remains challenging. The number of exosomes needed in humans is many times larger than that required in mice, and the low yield of exosomes is a major challenge that significantly impedes the relevant industrial and clinical translation [54]. Second, exosomes currently have multiple other extraction approaches, and different methods may have slight differences. Ensuring the stability of the inclusions during their extraction and their therapeutic effects on diseases remain to be explored [55].

## Conclusion

The collective findings of the present study demonstrate that HucMSC-Exos can regulate necroptosis through miR-874-3p to attenuate renal tubular epithelial cell injury and enhance repair. This study provides new therapeutic modalities and ideas for the treatment of AKI and the process of AKI to CKD transformation to mitigate renal damage.

## Supplementary Information

**Additional file 1.** Supplementary Figures.

**Additional file 2.** Supplementary Tables (Primers and antibodies informations).

## Data Availability

The data presented in this study are available on reasonable request from the corresponding authors.

## Abbreviations

HucMSC-Exo: Human umbilical cord mesenchymal stem cell exosome
AKI: Acute kidney injury
CKD: Chronic kidney disease
ESRD: End-stage renal disease
RIPK: Receptor-interacting protein kinase
MLKL: Mixed-lineage kinase domain-like pseudokinase
PGAM5: Phosphoglycerate mutase 5
Drp1: Dynamin-related protein 1
mtROS: Mitochondrial reactive oxygen species
mtDNA: Mitochondrial DNA
TNF: Tumor necrosis factor
UUO: Unilateral ureter obstruction
miRNA: MicroRNA
HE: Hematoxylin–eosin
PAS: Periodic acid–Schiff
HBSS: Hank’s balanced salt solution
UTR: Untranslated region
Co-IP: Co-immunoprecipitation
Kim 1: Kidney injury molecule 1
α-SMA: Alpha-smooth muscle actin
Mfn: Mitochondrial fusion
NC: Negative control
