# Supplementary material for: Human umbilical cord mesenchymal stem cell exosome-derived miR-874-3p targeting RIPK1/PGAM5 attenuates kidney tubular epithelial cell damage
Source: Cell Mol Biol Lett. 2023 Feb 7;28:12. doi: 10.1186/s11658-023-00425-0 (PMC9903493; doi:10.1186/s11658-023-00425-0)
Supplement: Supplementary file 1 — Additional file 1. Supplementary Figures. [file 11658_2023_425_MOESM1_ESM.docx]

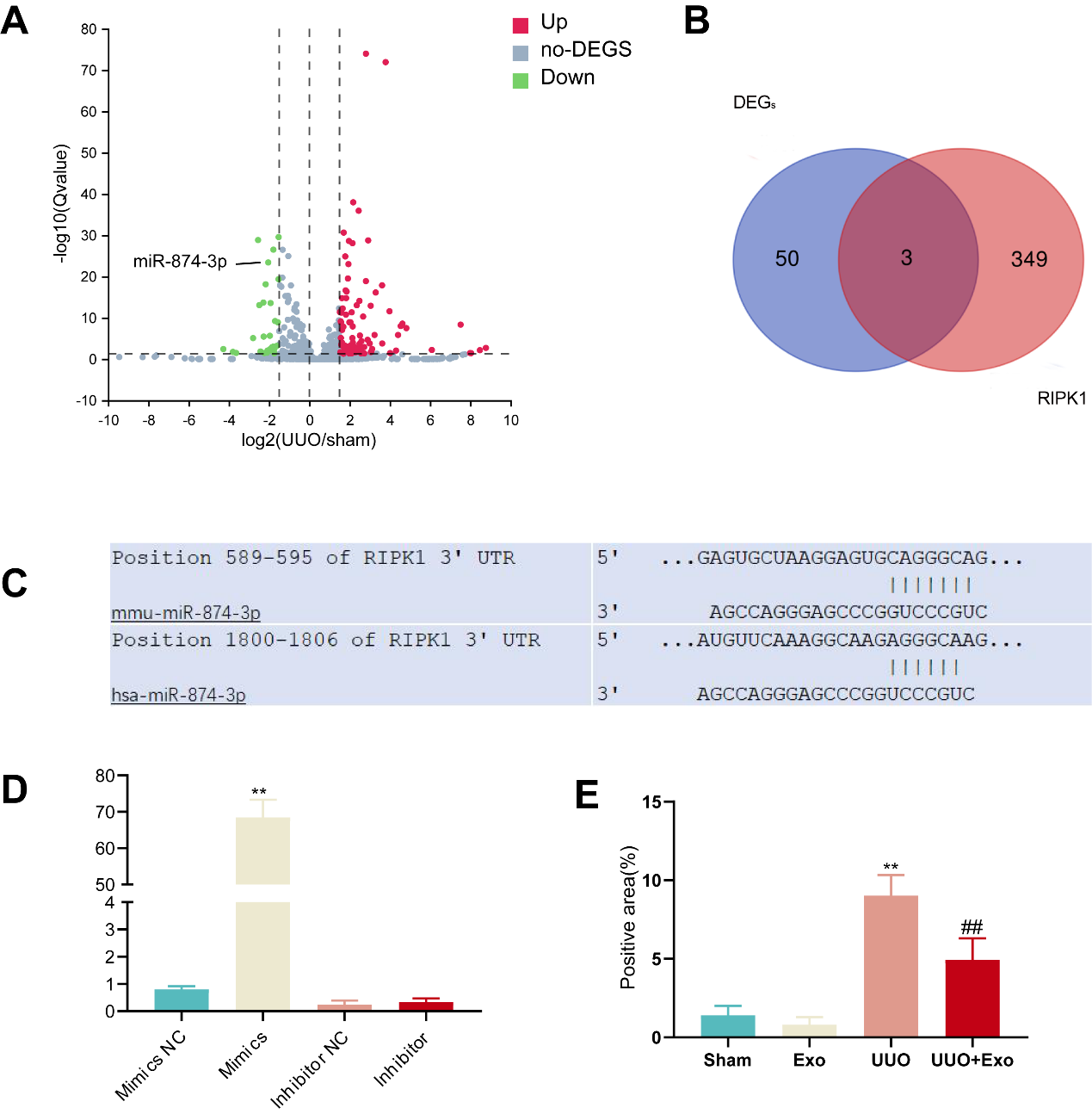


Supplemental Fig. 1 Screening for effector miRNAs and transfecting miR-874-3p mimics into HK-2 cells in vitro. (A) Analysis of differential miRNA expression in the kidneys of the UUO and Sham groups by transcriptome sequencing revealed a significant low expression of miR-874-3p in the UUO group; (B) Combine the low-expressed miRNAs in the UUO group of transcriptome sequencing and the miRNAs targeting RIPK1 in the TargetScan database, and take their intersection with the Venn diagram; (C) Predicted binding sites of mi874-3p to RIPK1 in human and mouse, respectively; (D) Transfection of miR-874-3p mimics/NC and inhibitor/NC in HK-2 cells , quantitative PCR analysis of miR-874 expression in each group, **p＜0.01, Compared to Mimics NC group; (E) Positive area of Masson stanning (%) *p< 0.05, **p< 0.01, compared with Sham group; ^#^p< 0.05, ^##^p < 0.01, compared with UUO group.


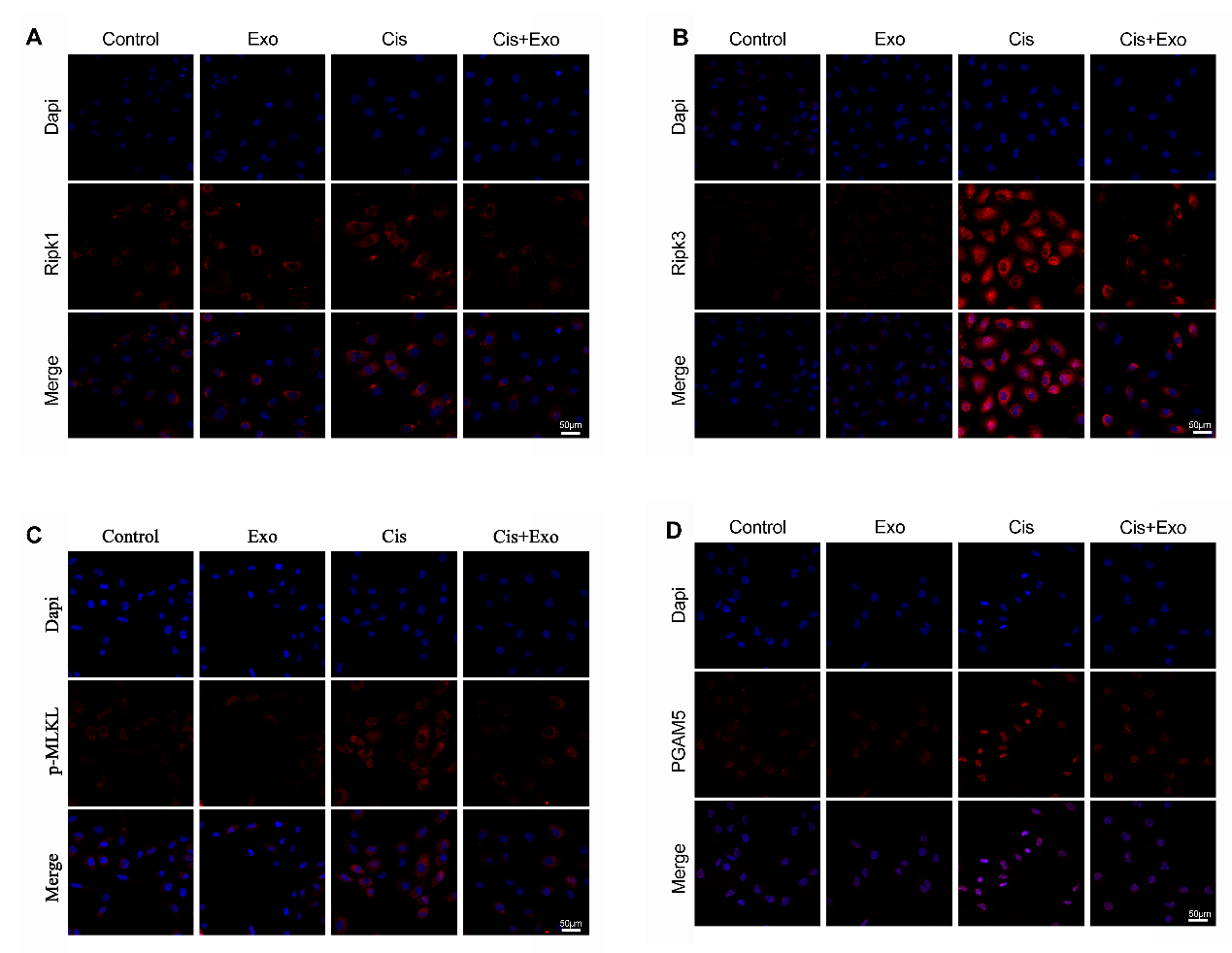


Supplemental Fig. 2 Immunohistochemistry detected of necroptosis related-markers in each group in vitro. (A) Immunohistochemistry detected of RIPK1; (B) Immunohistochemistry detected of RIPK3; (C) Immunohistochemistry detected of p-MLKL; (D) Immunohistochemistry detected of PGAM5.
