# Supplementary material for: Human umbilical cord mesenchymal stem cell exosome-derived miR-874-3p targeting RIPK1/PGAM5 attenuates kidney tubular epithelial cell damage
Source: Cell Mol Biol Lett. 2023 Feb 7;28:12. doi: 10.1186/s11658-023-00425-0 (PMC9903493; doi:10.1186/s11658-023-00425-0)
Supplement: Supplementary file 2 — Additional file 2. Supplementary Tables (Primers and antibodies informations). [file 11658_2023_425_MOESM2_ESM.docx]

Table1 MiR-874-3p interference sequence

| MiR-874-3p interference | Sequence (5’-3’) |
| --- | --- |
| MiR-874-3p mimics | 5’-CUGCCCUGGCCCGAGGGACCGA-3’ |
|  | 5’- UCGGUCCCUCGGGCCAGGGCAG-3’ |
| MiR-874-3p mimics NC | 5’-UUUGUACUACACAAAAGUACUG-3’ |
|  | 5’-CAGUACUUUUGUGUAGUACAAA-3’ |
| MiR-874-3p inhibitor | 5’- UCGGUCCCUCGGGCCAGGGCAG-3’ |
| MiR-874-3p inhibitor NC | 5’-CAGUACUUUUGUGUAGUACAAA-3’ |

Table2 Sequence-specific primers

| Primers | Sequence (5’-3’) |
| --- | --- |
| MiR-874-3p | Fwd: 5’-TATAACTGCCCTGGCCCGAG-3’ |
| U6 | Fwd: 5’-CTCGCTTCGGCAGCACATA-3’ |

The reverse qPCR primer was included in the kit.

Table3 Antibodies and their sources

| Antibody | Application | Company |
| --- | --- | --- |
| CD63 | WB(1:500) | Ab134045, Abcam, American |
| Alix | WB(1:1000) | 12422-1-AP, Proteintech, China |
| Tsg101 | WB(1:1000) | 28283-1-AP, Proteintech, China |
| Fibronectin | WB(1:1000), | 15613-1-AP, Proteintech, China |
| Vimentin | WB(1:1000),IHC(1:200) | R22775, Zenbio, China |
| α-SMA | WB(1:1000), IHC(1:200) | 380653, Zenbio, China |
| Kim1 | WB(1:1000), | Bs-2713R, Bioss, China |
| CollagenⅠ | WB(1:1000), IHC(1:200) | Bs-10423R, Bioss, China |
| RIPK1 | WB(1:1000), IHC,IF(1:200) | 17519-1-AP, Proteintech, China |
| RIPK3 | WB(1:1000), IHC,IF(1:200) | 17563-1-AP, Proteintech, China |
| MLKL | WB(1:1000), | 21066-1-AP, Proteintech, China |
| p-MLKL | WB(1:1000), IHC,IF(1:200) | PA5-105678, Invitrogen, American |
| PGAM5 | WB(1:500), | Sc-515880, Santa Cruz, American |
| Tomm20 | IF(1:200) | 11802-1-AP, Proteintech, China |
| Drp1 | WB(1:1000), | DF7037, Affinity, China |
| p-Drp1 | WB(1:1000), | DF2980, Affinity, China |
| Mfn1 | WB(1:1000), IHC(1:200) | 13798-1-AP, Proteintech, China |
| Mfn2 | WB(1:1000),IF(1:200) | 12186-1-AP, Proteintech, China |
| GAPDH | WB(1:5000), | 200306, Zenbio, China |
